# Costs and economies of scale in the accelerated program for prevention of mother-to-child transmission of HIV in Zimbabwe

**DOI:** 10.1371/journal.pone.0231527

**Published:** 2020-05-20

**Authors:** I. Ochoa-Moreno, S. Bautista-Arredondo, S. I. McCoy, R. Buzdugan, C. Mangenah, N. S. Padian, F. M. Cowan

**Affiliations:** 1 National Institute of Public Health, Cuernavaca, Mexico; 2 University of York, York, England, United Kingdom; 3 University of California, Berkeley, California, United States of America; 4 Centre for Sexual Health and HIV/AIDS Research, Harare, Zimbabwe; 5 Liverpool School of Tropical Medicine, Liverpool, England, United Kingdom; Universidad Loyola Andalucia Cordoba, SPAIN

## Abstract

**Background:**

Despite a growing body of literature on HIV service costs in sub-Saharan Africa, only a few studies have estimated the facility-level cost of prevention of Mother-to-Child Transmission (PMTCT) services, and even fewer provide insights into the variation of PMTCT costs across facilities. In this study, we present the first empirical costs estimation of the accelerated program for the prevention of mother-to-child transmission of HIV in Zimbabwe and investigate the determinants of heterogeneity of the facility-level average cost per service. To understand such variation, we explored the association between average costs per service and supply-and demand-side characteristics, and quality of services. One aspect of the supply-side we explore carefully is the scale of production—which we define as the annual number of women tested or the yearly number of HIV-positive women on prophylaxis.

**Methods:**

We collected rich data on the costs and PMTCT services provided by 157 health facilities out of 699 catchment areas in five provinces in Zimbabwe for 2013. In each health facility, we measured total costs and the number of women covered with PMTCT services and estimated the average cost per woman tested and the average cost per woman on either ARV prophylaxis or ART. We refer to these facility-level average costs per service as unitary costs. We also collected information on potential determinants of the variation of unitary costs. On the supply-side, we gathered data on the scale of production, staff composition and on the types of antenatal and family planning services provided. On the demand side, we measured the total population at the catchment area and surveyed eligible pairs of mothers and infants about previous use of HIV testing and prenatal care, and on the HIV status of both mothers and infants. We explored the determinants of unitary cost variation using a two-stage linear regression strategy.

**Results:**

The average annual total cost of the PMTCT program per facility was US$16,821 (median US$8,920). The average cost per pregnant woman tested was US$80 (median US$47), and the average cost per HIV-positive pregnant woman initiated on ARV prophylaxis or treatment was US$786 annually (median US$420). We found substantial heterogeneity of unitary costs across facilities regardless of facility type. The scale of production was a strong predictor of unitary costs variation across facilities, with a negative and statistically significant correlation between the two variables (p<0.01).

**Conclusions:**

These findings are the first empirical estimations of PMTCT costs in Zimbabwe. Unitary costs were found to be heterogeneous across health facilities, with evidence consistent with economies of scale.

## Introduction

Prevention of Mother-to-Child Transmission (PMTCT) is an integral component of HIV prevention programs in generalized epidemics. If implemented effectively, PMTCT has the potential to reduce HIV among infants [1], extend life expectancy and improve quality of life for HIV-positive women [2], and reduce HIV transmission to adult men [3,4]. The roll-out of PMTCT has already led to substantial gains globally–between 2000 and 2015, 1.6 million HIV infections among infants were averted, and pediatric deaths were reduced by 70% [5]. Unfortunately, the UNAIDS goal of virtual elimination of mother-to-child transmission (EMTCT) is far from complete; 150,000 infants were born with HIV in 2015 [5].

The ambitious target of EMTCT initially set for 2015 as a millennium development goal [6], requires programs to improve efficiency while maintaining quality [7–9]. Zimbabwe has an HIV prevalence of 13.5%, 1.3 million people were living with the disease in 2016 [10]. After expanding the PMTCT Program in 2014, 93% of the HIV infected pregnant women received antiretroviral therapy (ART) to prevent mother-to-child-transmission (MTCT) [10]. MTCT was estimated to account for 6.39% of all new HIV infections in children aged 0 to 14 years in 2015. Despite all efforts, only 54.9% of infants born to HIV-positive mothers received an HIV test in the first two months of life [11].

The literature evaluating efficiency and costs of HIV services has frequently focused on program- or national-level cost estimates, which often mask substantial heterogeneity at the clinic level. Although there is a growing body of literature on the per-client cost of HIV services in sub-Saharan Africa [12–21], only a handful of studies have examined the facility-level cost of PMTCT [13, 14, 19, 21], and among those, just a few used sample sizes larger than one or two facilities [13,14,19,21]. Thus, most of these studies estimated average costs without offering insights on the variation of the cost per service across facilities.

Examining variation across facilities as well as both the demand- and supply-side characteristics associated with it is critical to understand efficiency. For example, the ORPHEA study [22] examined supply-side determinants of unitary cost variation of PMTCT programs in four countries in sub-Saharan Africa: Kenya, Rwanda, South Africa, and Zambia. The authors found that scale (defined as the annual number of services produced) and the type of facility (hospital or health clinic), were strongly and significantly associated with unitary cost variation across sites [13], consistent with the existence of economies of scale–i.e., decreasing unitary costs as the scale increases.

The objective of this paper is twofold: 1) to estimate the facility-level total costs and the average cost per woman served (unitary costs) in the Accelerated National PMTCT Program. We measured services along two steps in the service cascade–HIV testing and counseling, and antiretroviral (ARV) prophylaxis or initiation of antiretroviral therapy (ART). 2) To explore determinants of unitary cost variability across facilities. We investigate three potential sources of variation. On the supply side, we explore the role of characteristics of the facilities, such as scale, the type of facility, the staff composition, location of the facilities, and availability of other related services nearby. On the demand side, we investigate the maternal HIV prevalence, MTCT rates, HIV free infant survival rate at 9–18 months, and uptake of HIV testing during pregnancy—all at the catchment area level. Finally, we also explore whether quality—as measured by the completeness of family planning, antenatal, and PMTCT services—is associated with unitary costs variation. We use data from a cross-sectional survey of 157 randomly selected facilities offering PMTCT services and their catchment areas, in five provinces in Zimbabwe.

## Methods

We analyzed 2013 data from the impact evaluation of Zimbabwe’s Accelerated National PMTCT Program. The project was funded by the Children’s Investment Fund Foundation together with Elizabeth Glaser Pediatric AIDS Foundation Zimbabwe. The impact evaluation combines cross-sectional surveys to determine the impact of Zimbabwe’s EMTCT program on MTCT as well as HIV-free survival among children aged 9 to 18 months. Previous results from this project have analyzed different aspects of the Program. For example, McCoy et al. (2015) examined the uptake of services and determined the factors associated with MTCT, maternal ART, and ARV prophylaxis [23]. Buzdugan et al. (2015) estimated HIV-free infant survival and MTCT rates [24], and Buzdugan et al. (2016) evaluated the impact of Option A on HIV-free infant survival and MTCT [25]. In this paper, we provide the baseline assessment of the efficiency of the PMTCT Program, using 2013 data from household and facility surveys, collected in 2014.

### Theoretical framework

In this analysis, we adopt a microeconomic lens to analyze efficiency. In the case of PMTCT services supplied by government facilities, scale (the annual number of PMTCT services provided) cannot be assumed to be exogenous–as is typically the case in the standard microeconomic model. The level of output produced by a health facility is determined as much by decisions made in the facilities, as by decisions made by patients. Health facilities choose not only how to allocate their resources, but also when to offer services, the quality of those services, and in general the type of experience they provide to patients. The results from those decisions in turn influence patients, who decide whether to demand those services or not, subject to their budget and other financial and non-financial constraints; and where to demand those services, constrained by the availability of nearby services. Thus, scale, measured as the number of services provided, is the result of decisions on the supply side by health providers, as well as decisions on the demand side by patients.

Two critical elements on the demand side, which may influence efficiency, are size and complexity. Higher demand potentially leads to an expansion of scale, which in turn is associated with lower unitary costs because fixed costs are distributed across more patients and also because human resources operate closer to full capacity (less idle time). This gain in efficiency as a result of larger scale is known as economies of scale. On the other hand, populations with more complex needs may increase costs. For example, the most expensive drug combinations that include more than three different elements are recommended only to patients in the most advanced stage of the disease [26], or high-risk pregnancies referred to hospitals require more and costlier resources, including staff, drugs, and laboratory tests.

In the analysis presented in this paper, we used both types of characteristics of the demand–size and complexity, and we use the size of population on clinics’ catchment areas as an instrument for the endogenous measure of scale. For proxies of demand complexity, we used maternal HIV prevalence; rate of mother-to-child transmission of HIV; rate of HIV-free infant survival; and uptake of HIV testing during pregnancy.

### Description of the intervention

In 2010, the World Health Organization issued guidelines for EMTCT based on the CD4 count of HIV positive mothers. CD4 T-cells are a type of white cell part of the immune system that fights bacteria, viruses, etc. The CD4 count is a test that measures the number of T-cells to assess the immune system’s health. HIV-infected pregnant women with immune damage of less than 350 CD4 cells, were eligible for lifelong antiretroviral treatment (ART). On the other hand, pregnant women ineligible for ART (CD4>350) were recommended to receive one of two options of prophylactic regimens: Option A consisted of starting at 14 weeks of pregnancy through 7 days postpartum; or Option B, an ARV preventive regime beginning at 14 weeks of pregnancy and continuing until weaning [27]. In 2013 and 2015, WHO revised these guidelines and rolled out Option B+, which recommended that all HIV infected pregnant women should receive lifelong ARV treatment regardless of CD4 count starting as soon as diagnosed [28, 29].

The Accelerated National PMTCT Program was implemented in 2011 by Zimbabwe's Ministry of Health and Child Care (MoHCC) based on PMTCT Option A from WHO's 2010 guidelines. Zimbabwe then switched to Option B+ in late 2013. [27, 30, 31]. Thus, at the time covered by our surveys (2012–2013), pregnant women were not exposed to Option B+. Under Option A, non-eligible HIV-positive women received ARV prophylaxis starting at 14 weeks of pregnancy through 7 days postpartum. HIV-infected pregnant women with CD4<350, regardless of symptoms, were eligible to receive lifelong ART. The recommended ARV regimen was Zidovudine (AZT) twice daily for the mother, and prophylaxis with either AZT or Nevirapine (NVP) for six weeks after birth if the mother was not breastfeeding the infant. If the infant was breastfeeding, daily NVP infant prophylaxis continued for one week after the end of the breastfeeding period [27, 30].

### Sampling strategy

The sampling strategy followed a two-stage process. First, we randomly selected 157 out of 699 health facilities from five provinces that provided PMTCT services for the entirety of 2013 in Zimbabwe: Harare, Mashonaland West, Mashonaland Central, Manicaland, and Matabeleland South. These provinces were selected to include three of the four largest cities in Zimbabwe, rural communities with high and low HIV prevalence, representation of both major ethnic groups in Zimbabwe (Shona and Ndebele), and areas where detailed monitoring-and-evaluation data were being collected [24]. Second, in each catchment area–defined as a neighborhood of a 10-km radius around each facility, we identified 21,205 eligible pairs of mothers/caregivers aged 16 or older and infants (born 9–18 months before the survey). We randomly selected a fraction of all mother-infant pairs with the aim of recruiting 50 from each catchment area to participate in the study. Overall, 9,087 mother-infant pairs did. The sampling strategy was previously described in more detail [23, 24, 25, 32].

We estimated a sample size of 157 PMTCT clinic catchment areas. We expected to identify an average of 190 infants aged 9–18 months per catchment area of PMTCT clinic, assuming 10.5 living infants aged 9–18 months per 100 households and approximately 1,800 households per catchment area of a PMTCT clinic. If we identified all eligible infants and enrolled 1 in every four eligible infants, this would result in 47.5 living infants aged 9–18 months per catchment area. Our estimated sample size for the baseline community survey is approximately 7,800 infants (7,442 alive and 353 deceased) and their mothers or caregivers from 157 PMTCT catchment areas. However, those initial sample size calculations were revised upwards after incorporating new data on variability in the number of eligible mother-infant pairs and the underlying HIV prevalence across catchment areas. The overall size of the population recruited increasing from an estimated 7,800 to 9,087.

### Data collection and measurement

#### Facility-level characteristics

The survey team administered a short questionnaire in each of the 157 health facilities. All data were collected on paper by a trained data collector and later entered into an Access™ database by a data entry operator. Researchers conducted periodic reviews of the data for completeness and consistency.

We collected data on the type of facility visited, according to Zimbabwe’s classification of health centers. Our sample included ten different types of health facilities; however, for the analysis, we grouped them into hospitals and non-hospitals (See S 1 for a detailed description of the different types of facilities). PMTCT services costs in hospitals tend to be higher. One important reason is that hospitals are more complex facilities, given that they provide not only outpatient but also inpatient services. Hospitals also tend to include more specialized, therefore higher paid medical staff. Because of these reasons, one hypothesis regarding our study is that hospitals are less flexible in terms of allocation of resources compared to clinics. This lack of adaptability could decrease efficiency and increase costs.

#### Costs

We adopted the perspective of service providers on the analysis. Therefore, we did not measure patients’ expenditures, such as out-of-pocket fees or transportation expenses. Moreover, only 4% of the women in the sample declared to have paid for PMTCT services.

We used a retrospective microcosting approach to measure monthly quantities and prices of three essential input categories: personnel, ARV drugs, and HIV tests kits. These categories comprise the largest share of the total costs of PMTCT services, according to previous studies [13, 14, 33]. We did not include other inputs involved in the provision of PMTCT, which typically represent less than 10% of the total costs [13, 14, 33], such as capital costs, training, supervision, and other recurrent costs.

We valued all inputs at market prices, including donations, adopting an economic rather than a financial costing approach. Given that all the units in our sample are government clinics, there is no variation in input prices or salaries across facilities. The Ministry of Health centrally establishes wages based on cadre categories and purchases all essential inputs (drugs and tests) through centralized procurement processes. Thus, the variation in unitary costs across facilities reflects differences in efficiency; the ability of clinics to produce services, given the resources at their disposal and given the demand characteristics they face.

We collected data on the total annual outputs produced along two steps in the PMTCT service cascade–HIV testing and ARV/ART initiation. When these data were not available at the facilities, we collected them from the district health information system.

We also assessed the time allocation of personnel providing PMTCT services through interviews with five randomly selected providers per facility, in which we asked them to report the time they spent working on PMTCT every day of the previous week. Self-reported time allocation to specific tasks has been found to overestimate effort allocated to particular services [34]. We attempted to minimize this potential bias in three ways. First, instead of asking about a “typical week” or a “typical year” which is a common wording for these questions, we asked about the “last week”; this would spare the respondent any mental calculation and make it easier to remember. Secondly, we clarified the purpose of the questions, which was not part of a performance evaluation. Finally, we assured respondents that the information they provided would be used only in statistical analyses at the facility level.

We categorized staff in three types of nurses (primary, general, and sisters in charge) and one broad category of health personnel which includes counselors, health promotion officers and other support staff (S 2). "Primary care nurse" was the category with the lowest average salary, and the second most common in the sample, after "general nurse."

#### Quality

Additionally, we collected information on the scope and comprehensiveness of PMTCT and other related maternal services provided at the facilities, as a proxy for quality. Process quality, which measures the extent to which providers follow the processes outlined or explicitly listed in official guidelines, has been used previously as an indicator of the quality of health care [35]. For example, Marley et al. (2004) found that process quality is as good as clinical quality in predicting patient satisfaction in hospitals in the U.S. [36]. Rademakers (2011) found that processes followed in hospitals in the Netherlands explained most of the variation of the patients' evaluation of quality of surgery and other interventions [37]. Meehan (1997) assessed the quality of care for Medicare patients hospitalized with pneumonia and found an association between the process of care and mortality [38].

There is also evidence from less affluent countries; Das and Gertler (2007) document practice quality on six different studies in five low- and middle-income countries. They provide evidence on the large effect of process quality on health outcomes compared to those of availability or structural quality [34]. Das and Hammer (2014) argue that access to healthcare is not the main problem in low-income countries anymore [35]. They provide evidence suggesting that process quality to be the real issue.

We collected information on the availability of services offered at the facilities as a proxy for process quality. Specifically, we asked which services they provided out of a comprehensive list of family planning, ANC and ART/ARV prophylaxis services recommended by the government. For example, whether contraception services were offered during antenatal visits, after labor and delivery, during postnatal care visits, and during child immunization visits, and constructed a variable index for contraception services based on the number of recommended care practices undertaken in the facility. We computed similar variables for antenatal services and ARV prophylaxis or ART (see S 2 for the complete list of recommended processes used for each indicator).

#### Demand-side characteristics

We also collected data on some characteristics of the demand as proxies for size and complexity. We surveyed mothers about their HIV status and estimated the maternal HIV prevalence; the rate of mother-to-child transmission of HIV; the rate of HIV-free infant survival; and the uptake of HIV testing during pregnancy. Finally, we asked each facility for the estimated total size of the population they served. All of these variables refer to the catchment area level.

The survey team administered a household-level survey in 2014 to the selected sample of 9,087 mother-infant pairs. Mothers or caregivers 16 years old and older and their infants born between 9 and 18 months before the interview (alive or deceased), were eligible to participate in the study. The data enumerator sought informed consent to complete the questionnaire and to collect dried blood samples (DBS) for HIV testing from the mother and her eligible infant. The mother could consent to participate in both, neither, or either the questionnaire and DBS.

The questionnaire was administered using a Personal Digital Assistant (PDA) and captured the mother’s demographic characteristics, her experience with ANC and HIV testing, and more specifically, her experience with ANC during the pregnancy for the eligible child [31]. Finally, all living biological mothers and infants provided DBS for HIV testing.

### Analysis

#### Estimation of costs

We calculated the total annual costs of PMTCT services for each facility as the sum of personnel and recurrent inputs costs used in 2013, as follows:

$$TC_{j}=\sum h_{ij}*w_{i}+\sum x_{jk}*p_{k}$$

Where TC_j_ denotes total costs of facility j. Personnel costs were estimated by the sum of the number of hours h_ij_ each type of personnel i in a facility j worked on PMTCT services, multiplied by the hourly wage w_i_ corresponding to provider type i. Recurrent costs (ART drugs and HIV test kits) were calculated as the sum of the k number of goods x_jk_ multiplied by their prices p_k_.

Then, the facility-level average unitary costs per output, AC_jl_ along the cascade were defined as:

$$AC_{jl}=\frac{TC_{j}}{q_{jl}}$$

Where q_jl_ is the 2013 annual number of outputs produced by facility j along the cascade indicator l, where l = 1 for number of pregnant women tested, l = 2 for HIV positive women on ARV prophylaxis or treatment.

The objective of this costing approach is to explore the efficiency of the PMTCT program by looking at the heterogeneity of "unitary costs" across implementers. This approach provides insight on the efficiency of the program at the facility level and across the service cascade.

#### Variation of unitary costs

We explored the facility-level variation of the unitary costs of PMTCT at two steps of the service cascade: HIV testing and ARV prophylaxis or ART. First, we present the dispersion of average unitary costs by type of facility to describe the variation across facilities. Then, we explore three potential determinants of the heterogeneity in unitary costs. On the supply side, we investigated the role of scale, staff categories, and facility type. On the demand side, we included in the analysis the prevalence of maternal HIV, the rates of mother-to-child transmission of HIV and HIV-free infant survival at 9–18 months, and the levels of uptake of HIV testing during pregnancy. Finally, we analyzed the role of the quality of services.

#### Analytic strategy

We were interested in identifying how supply- and demand-side characteristics influence the variation of unitary costs across facilities, and in particular the role of the scale of PMTCT services. However, a simple linear regression could yield a biased parameter due to endogeneity bias since there may be unobservable or omitted characteristics simultaneously explaining costs and scale. To minimize this potential bias, we apply a two-stage least square model using demand size, measured by the size of the population at the catchment-area level, as an instrumental variable (IV). With this approach, we estimated first, the association between demand size and scale; and, second, the effect of scale and other supply-side characteristics on the unitary costs of PMTCT services.

The second stage regression model is specified as follows:

$$y_{i}=\alpha_{0}+\pi x_{i}^{*}+\gamma_{s}s_{si}+\delta_{d}d_{di}+\eta_{q}q_{qi}+\varepsilon_{i}$$ (1)

Where y_i_ represents the log-transformed unitary costs of PMTCT services (for each step of the cascade): the average cost per pregnant woman tested for HIV in facility i, or the average cost of an HIV-infected pregnant woman on ARV prophylaxis or ART in facility i. Scale is the endogenous variable represented by x_i_, s_si_ is a vector of s supply characteristics including a binary variable for type of facility (hospital or non-hospital), the proportion of primary care nurses with respect to the other types of staff; d_di_ is a vector of d demand-side characteristics, q_qi_ is a vector of q process quality variables; and ε_i_ is the residual term.

In the first stage (Eq 2), we regress the endogenous variable on the exogenous variables in the model including our instrument, the log-transformed size of the population in the catchment area (z_i_) to obtain adjusted values of x (x*) and use them in the second stage of the analysis (Eq 1) to obtain unbiased estimates of y.

$$x_{i}^{*}=z_{i}\pi_{z}+\pi_{0}+\pi_{1s}s_{si}+\pi_{2d}d_{di}+\pi_{3q}q_{qi}+v_{i}$$ (2)

Although the validity of an instrumental variable cannot be fully statistically tested, we computed the first stage F-statistic to verify the condition of the Stock-Yogo critical values [39]. To test for endogeneity, we implemented the Durbin-Wu-Hausman test with the null hypothesis of exogeneity. However, we also verified the two conditions for the instrumental variable model to be valid. First, we descriptively examined the association between the instrumental variable ‘population size’ and the endogenous variable ‘scale’ of PMTCT services production (see S3 and S4 Files). We found a meaningful and statistically significant association. Secondly, we explored the association between costs and the size of the population and found no statistically significant correlation, suggesting the instrumental variable (population size) influences the dependent variable only through scale. We acknowledge that different channels could also be associated with population size and costs. For example, more populated areas concentrate more skilled providers which could translate into higher staff costs or more efficient use of resources. There appears to be a low correlation between higher skilled providers and costs; however, this is not statistically significant. Nevertheless, we have addressed this possible source of bias by controlling for staff composition in all our regressions. Overall, our results are consistent with our assumption that the area population affects costs only through scale, rendering validity to our approach.

In addition to the IV model, we estimated two naïve OLS models, one for each of the two stages of the PMTCT cascade. These OLS models regressed log-transformed costs on the log-transformed scale and controlled for the same supply-side characteristics and quality/completeness, as the IV models. We used robust standard errors and province fixed effects in all regressions.

#### Human subjects research

This research was approved by the Institutional Review Board of the University of California Berkeley.

## Results

### Costs of PMTCT services

Table 1 shows descriptive statistics of unitary costs, supply- and demand-side, and quality indicators. From the sample of 157 health facilities, 17% were hospitals. Approximately half of the staff were primary care nurses. On average, health facilities offered three out of four recommended ARV prophylaxis or treatment practices (S2 File), 5.5 out of six ANC services, and ten out of 12 contraceptive services. The average annual number of PMTCT clients tested per facility was 305 (median 186), and the yearly average number of HIV-positive women receiving ART or ARV prophylaxis for MTCT per facility was 33 (median, 19). The average annual total cost of PMTCT services was $16,821 (median, $8,920) in153 health facilities with complete information. Total yearly costs varied according to the type of facility; in hospitals, the average was $20,786, and in non-hospitals, it was $16,162.

**Table 1:** Description of the sample. Annual facility-level costs, scale and other supply-side characteristics.

	N	mean	sd	min	p50	max
Supply-side characteristics						
Proportion of hospitals	154	0.17	0.37	0	0	1
Proportion of staff that are primary care nurses	154	0.49	0.31	0	0.5	1
Proportion of facilities in urban areas	157	0.05	0.22	0	0	1
Catchment areas intersect	150	0.13	0.33	0	0	1
Demand-side characteristics						
Total population by facility	149	10,777	15,621	300	7,589	130,657
Maternal HIV prevalence	153	14.75	7.35	0	14.29	37.33
MTCT, 9–18 months	151	5.05	8.67	0	0	50
HIV-free infant survival, 9–18 months	151	94.90	8.69	50	100	100
Uptake of HIV testing during pregnancy	153	92.62	7.25	54.76	94.34	100
Quality/Completeness*						
ARV prophylaxis/ART services offered by facility	160	3.01	1.30	0	4	4
ANC services offered by facility	160	5.53	1.07	0	6	6
Contraception services offered by facility	160	10.03	2.20	0	11	12
Scale of production						
Number of women tested for HIV	137	305	727	0	186	8,365
Number of women on ARV prophylaxis/ART	137	33	49	0	19	319
Total costs (USD)						
Per facility	153	16,821	41,262	1,128	8,920	432,998
Per hospital	24	20,786	16,012	5,922	15,786	74,208
Per non-hospital health facility	128	16,162	44,575	1,128	8,422	432,998
Average unitary costs						
Per woman tested for HIV	135	80	83	12	47	528
Per woman on ARV prophylaxis/ART	127	786	892	61	420	5,018

*Quality/completeness variables are calculated as the number of recommended practices offered in a facility. (See S1 File)

The average unitary cost per pregnant woman tested was $80 (median, $47), and the average cost per HIV-infected pregnant woman receiving ART or ARV prophylaxis was $786 (median, $420) per year. The former ranged from $12 to $528, and the latter from $61 to $5,018.

**Fig 1:**
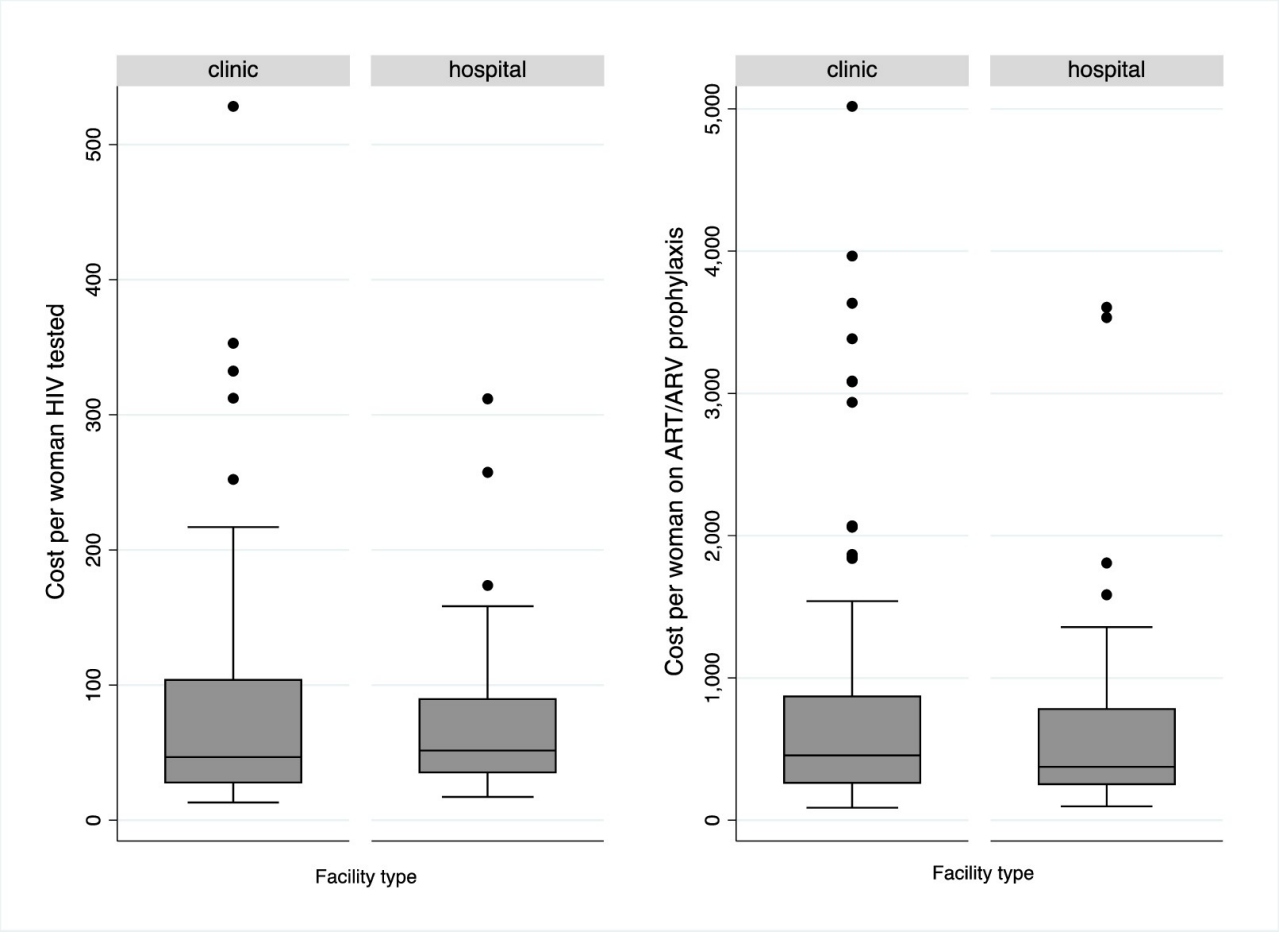
Dispersion of unitary costs by facility type.

This variation in unitary costs across facilities is further illustrated in Fig 1. There are substantial differences in the dispersion of costs between hospitals and non-hospitals. We find variability of two and three orders of magnitude across facilities in both the cost per woman tested and the cost per woman on ARV/ART, regardless of facility type.

**Fig 2:**
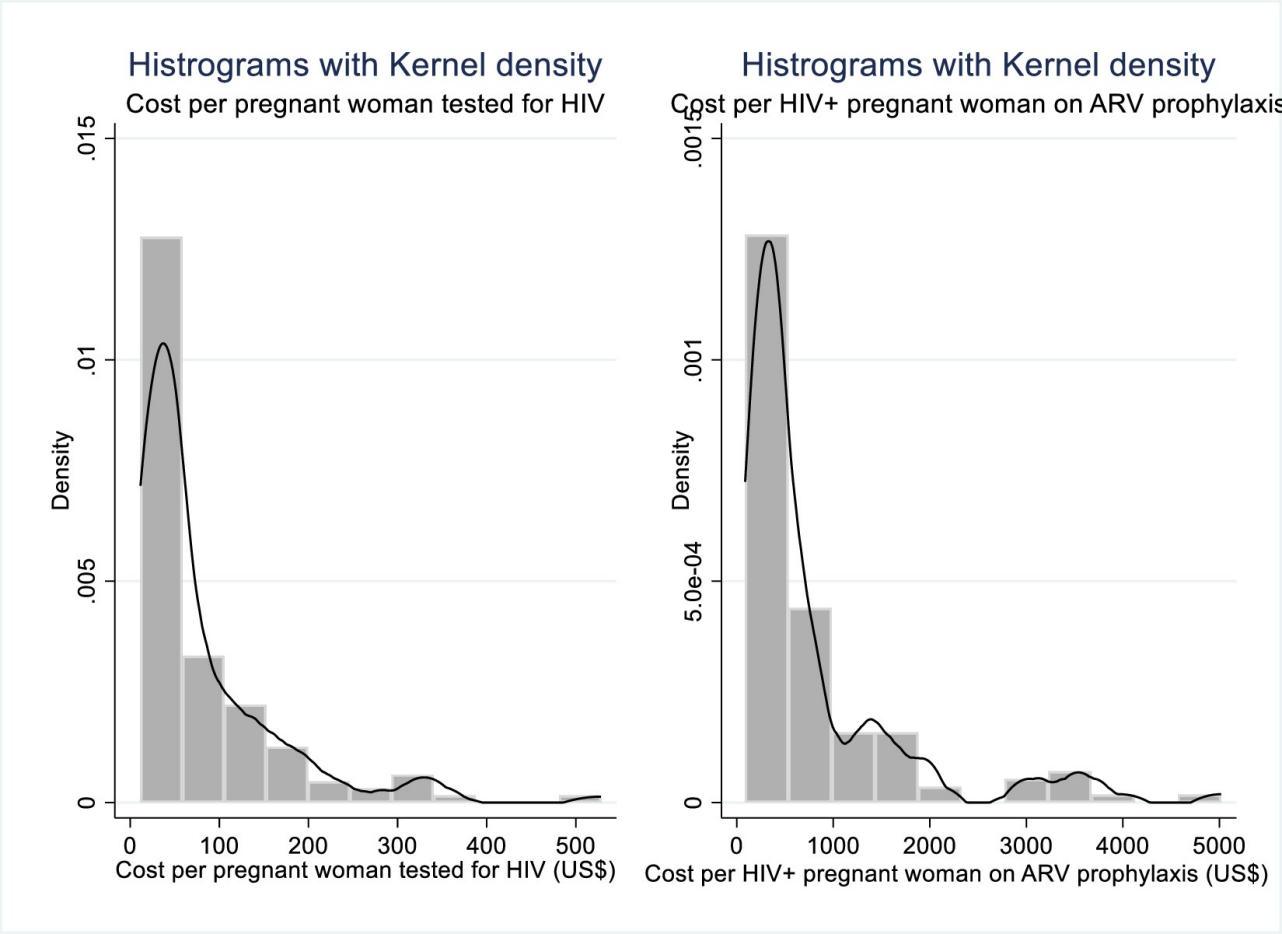
Histograms of unitary costs.

Healthcare costs data is usually skewed. To implement linear regression models, we transformed the data to natural logarithms and decrease skewness. This kind of transformation is a common approach in applied econometrics. Fig 2, illustrates the distribution of the unitary costs.

### Two-stage least squares estimation of the determinants of unitary costs

Table 2 shows the results of a two-stage least squares (2SLS) model estimation of the impact of scale on unitary costs, with the size of the population in the catchment area as an instrumental variable. Columns (1) and (2) present the results of the naïve OLS models. Columns (3) and (5) show the first-stage estimates of both 2SLS models, which show a significant association between the total population in the catchment area and scale. For every 10% increase in population size, there is an average 6.6% increase in the number of women tested, and an average 6% increase in the number of women on ART/ARV prophylaxis.

**Table 2:** OLS and 2SLS estimations of the determinants of unitary costs along two steps in the PMTCT service cascade.

	(1)	(2)	(3)	(4)	(5)	(6)
	Cost per woman tested (ln)	Cost per woman in ART (ln)	Women tested	Cost per woman tested	Women in ART	Cost per woman in ART
VARIABLES			1st	2nd	1st	2nd
Supply side characteristics						
Scale (ln)	-0.61***	-0.73***		-0.58***		-0.42**
	(0.07)	(0.08)		(0.11)		(0.18)
Hospital (0,1)	0.43**	0.36*	0.32*	0.42**	0.55**	0.14
	(0.17)	(0.18)	(0.19)	(0.17)	(0.26)	(0.22)
Primary care nurse (%)	-0.40*	-0.40	-0.18	-0.46**	-0.56	-0.27
	(0.23)	(0.28)	(0.28)	(0.20)	(0.36)	(0.27)
Urban area	0.53	1.32***	0.69**	0.18	0.45	0.76
	(0.34)	(0.47)	(0.31)	(0.32)	(0.54)	(0.51)
CD4 testing services within catchment area	-0.31**	-0.37**	-0.42**	-0.29**	-0.20	-0.31*
	(0.14)	(0.15)	(0.16)	(0.13)	(0.19)	(0.16)
Demand side characteristics						
Maternal HIV prevalence	0.008	0.001	-0.008	0.01	0.01	0.001
	(0.008)	(0.008)	(0.009)	(0.008)	(0.01)	(0.009)
MTCT, 9–18 months	-0.04	0.01	0.02	-0.09***	0.18**	-0.07
	(0.05)	(0.08)	(0.03)	(0.03)	(0.07)	(0.08)
HIV-free infant survival, 9–18 months	-0.05	0.006	0.02	-0.09***	0.19***	-0.08
	(0.05)	(0.08)	(0.03)	(0.03)	(0.07)	(0.08)
Uptake of HIV testing during pregnancy	0.009	0.003	0.009	0.009	0.02**	0.0002
	(0.007)	(0.008)	(0.008)	(0.007)	(0.01)	(0.01)
Process quality						
Offers contraception services	0.009	-0.006	0.02	-0.01	0.08	-0.06
	(0.04)	(0.05)	(0.05)	(0.04)	(0.06)	(0.06)
Offers ARV prophylaxis	0.04	0.18	0.25***	0.01	0.01	0.09
	(0.09)	(0.14)	(0.09)	(0.10)	(0.12)	(0.13)
Offers ANC services	0.01	0.009	0.00005	0.01	0.01	0.01
	(0.04)	(0.04)	(0.03)	(0.03)	(0.03)	(0.03)
Instrument						
Population in catchment area			0.66***		0.60***	
			(0.09)		(0.16)	
Constant	11.53*	6.78	-5.76	16.36***	-24.10**	15.59*
	(5.86)	(8.43)	(3.61)	(3.87)	(7.35)	(8.57)
Observations	130	122	127	127	118	118
Adj R-squared	0.50	0.47	0.53	0.59	0.38	0.49
Robust IV F-stat				47.21		13.94
Durbin pval				0.51		0.04

Standard errors in parentheses

*** p<0.01,

** p<0.05,

* p<0.1

Total population size in the catchment area satisfies the Stock-Yogo test for weak instruments as the F statistic for significance of the instruments in the first stage exceeds 10. The Durbin-Wu-Hausman test for over-identifying restrictions is satisfied only for cost per woman in ART but not for cost per woman tested implying that scale is not endogenous in regression 4 and OLS model 1 is valid. Table 2 provides both the F and DWH statistics.

**Fig 3:**
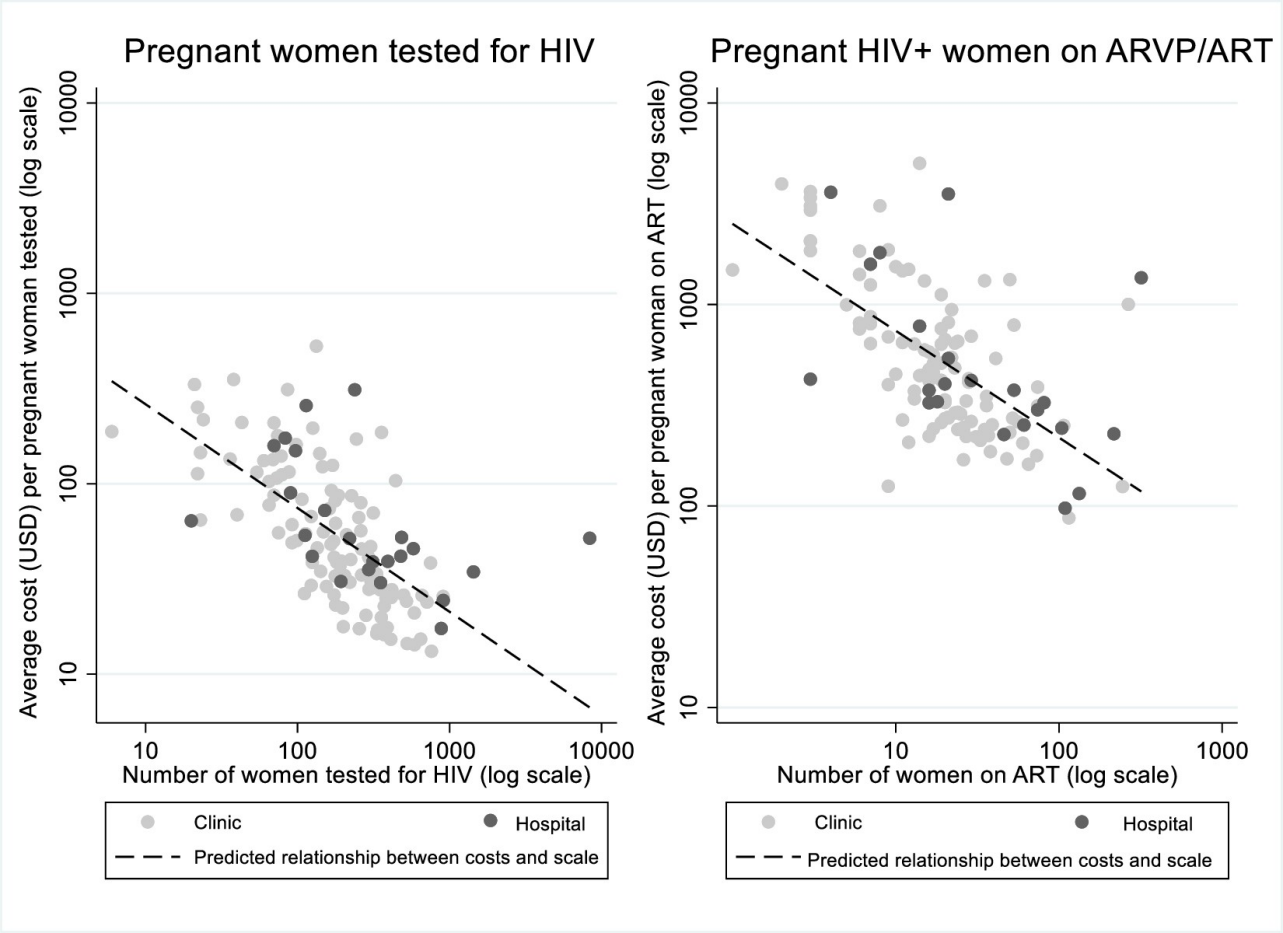
Unitary costs along the cascade and scale.

Second-stage estimates of the 2SLS model show a strong negative relationship between scale and unitary costs—Columns (4) and (6)—with slightly smaller coefficients than those from the naïve OLS estimations, which illustrates the relatively small magnitude of the bias introduced by the naïve models. According to the 2SLS results, an increase of 10% annual number of women served translated into an average 5.8% decrease in the cost per woman tested, and a 4.2% decrease in the cost per woman initiated on ART/ARV. Fig 3 illustrates the inverse relationship between costs and scale graphically.

Our identification strategy assumes that size of the population at the catchment area is a valid instrument, which means it only affects costs through scale. In order to test this assumption, we consider other paths through which population size may affect costs. For example, whether a catchment area is an urban area or not may affects costs. We tested this possibility and found that urbanicity and HIV test costs or ART costs are not correlated (0.01 and -0.01 respectively). Another reason for population size influencing costs other by scale could be that health professionals prefer to live in more populated areas, most likely urban areas, so there might be more competition to be relocated there. This may cause more concentration of higher skilled health providers in more populated areas which may increase the cost of health services. We explored this hypothesis and found that facilities in more populated areas have higher concentration of high skilled health providers (midwives 0.22, senior registered general nurses 0.25); and low concentration of low skilled providers (nurse aids -0.32). However, there is no association between proportion of skilled workers in the facility and costs (S4 File).

We further explore the relationship of costs and scale with non-linear OLS models. In Table 3 we present the linear OLS model previously used and add a quadratic and cubic model. Column (3) suggests a cubic relationship between cost per woman tested and scale. However, columns (4) to (6) do not seem to indicate nonlinear relationship between cost per woman in ART and scale. Columns (4) and (5) show a strong linear relationship between cost and scale, while Column (6) shows a weak relationship of a higher degree polynomial.

**Table 3:** Non-linear OLS models.

	(1)	(2)	(3)	(4)	(5)	(6)
	Cost per woman tested (ln)	Cost per woman tested (ln)	Cost per woman tested (ln)	Cost per woman in ART (ln)	Cost per woman in ART (ln)	Cost per woman in ART (ln)
VARIABLES						
Supply side characteristics						
Scale (ln)	-0.58***	-0.42	3.19***	-0.72***	-1.05***	0.19
	(0.07)	(0.46)	(0.76)	(0.07)	(0.37)	(0.64)
Scale squared (ln)		-0.01	-0.72***		0.06	-0.42*
		(0.04)	(0.13)		(0.06)	(0.23)
Scale cubic (ln)			0.04***			0.055**
			(0.007)			(0.02)
Hospital (0,1)	0.39**	0.40**	0.35*	0.33*	0.29	0.23
	(0.18)	(0.18)	(0.17)	(0.18)	(0.18)	(0.18)
Primary care nurse (%)	-0.36	-0.37	-0.32	-0.34	-0.25	-0.31
	(0.23)	(0.23)	(0.22)	(0.27)	(0.30)	(0.31)
Urban area	0.45	0.50	0.16	1.26**	1.00**	0.68*
	(0.34)	(0.33)	(0.27)	(0.48)	(0.46)	(0.40)
Demand side characteristics						
Maternal HIV prevalence	0.009	0.01	0.01	0.001	-0.001	-0.001
	(0.007)	(0.007)	(0.007)	(0.008)	(0.008)	(0.008)
MTCT, 9–18 months	-0.04	-0.03	-0.12***	0.01	-0.03	-0.09
	(0.05)	(0.05)	(0.04)	(0.08)	(0.08)	(0.07)
HIV-free infant survival, 9–18 months	-0.05	-0.04	-0.12***	0.009	-0.03	-0.09
	(0.05)	(0.05)	(0.03)	(0.08)	(0.08)	(0.07)
Uptake of HIV testing during pregnancy	0.008	0.008	0.008	0.001	0.002	0.002
	(0.007)	(0.007)	(0.006)	(0.008)	(0.008)	(0.007)
Process quality						
Offers contraception services	-0.001	0.001	0.007	-0.02	-0.02	-0.03
	(0.04)	(0.04)	(0.04)	(0.05)	(0.05)	(0.05)
Offers ARV prophylaxis	0.007	0.001	-0.06	0.16	0.15	0.13
	(0.10)	(0.10)	(0.09)	(0.14)	(0.14)	(0.14)
Offers ANC services	0.009	0.012	0.02	0.003	-0.003	-0.004
	(0.03)	(0.03)	(0.03)	(0.04)	(0.04)	(0.04)
Constant	11.38*	10.21*	12.40***	6.40	11.64	17.20**
	(5.88)	(5.89)	(4.45)	(8.54)	(9.09)	(8.11)
Observations	130	130	130	122	122	122
R-squared	0.49	0.48	0.53	0.46	0.46	0.48

Robust standard errors in parentheses

*** p<0.01,

** p<0.05,

* p<0.1

Results from Tables 2 and 3 suggest that there is a linear relationship between cost per woman on ART and scale that is endogenous and can be instrumented with population size at the catchment area level. Moreover, there is a polynomial relationship of degree three between cost per woman tested and scale.

The data also confirmed the relationship between personnel categories and unitary costs; with lower costs linked to a higher proportion of primary care nurses. We found a statistical association between the type of facility and lower costs per woman tested. There was no significant difference in unitary costs per woman on ART between hospitals and non-hospitals, and we found no association between process quality of reproductive and maternal services and unitary costs.

On the demand side, we found a significant association between maternal HIV prevalence and the proportion of women tested for HIV in prenatal care with higher HIV testing costs.

## Discussion

Using data from a cross-sectional survey of 157 randomly selected health facilities offering PMTCT services in Zimbabwe, in this paper we presented the first empirical estimations of the PMTCT program costs along the service cascade in Zimbabwe, adopting the perspective of the government. We found that the average annual total cost of PMTCT per facility was $16,821 (median, $8,920). The type of facility is a significant predictor, with an average of $20,786 in hospitals and $16,162 in non-hospitals. The average annual cost per service was $80 per pregnant woman tested for HIV (median, $47), and $786 per HIV-infected pregnant woman on ARV prophylaxis or ART (median, $420).

Our results are comparable to previously published estimates. In a study conducted in four African countries in 2013 [13], unitary costs ranged from $18 per pregnant woman tested in Rwanda to $89 in South Africa; and from $ 704 per woman in antiretroviral prophylaxis in South Africa to $ 2,314 in Rwanda. A similar study conducted in Nigeria [14], found that the facility-level unitary costs per woman tested, per HIV-positive woman diagnosed, and per HIV-positive woman on prophylaxis were $46, $2,932, and $3,647, respectively. In a systematic review of HIV treatment and PMTCT costs for low and middle-income countries [12], the median ART cost per patient per year in low-income countries was $792; while, for middle-income countries, the median was $932.

A unique feature of our study is the large sample size of facilities included in the analysis, which allowed us to analyze the technical efficiency of PMTCT service provision in Zimbabwe. Our results demonstrate the high variability of facility-level unitary costs, a finding consistent with previous research. For example, a study that identified significant heterogeneity in PMTCT unitary costs in Kenya, Nigeria, Rwanda, South Africa, and Zambia with sample size similar to ours, and suggested potential gains from more efficient delivery of services [13, 14].

In this study, we explored the relationship between supply-side characteristics, demand size and complexity, and unitary costs. Scale of services was the primary predictor of unitary cost variability, a link that has been documented previously [13, 14, 21, 40]. A review of economic methodologies that study the relationship between HIV/AIDS intervention costs and scale found that scale variation explains between 26% and 70% of cost variation across locations for similar interventions [40]. In Nigeria, a study found negative associations between scale and costs, implying decreasing rates of economies of scale [14]. Another study on six types of HIV prevention interventions in five low and middle-income countries found that efficiency increased with scale across all countries and interventions [21]. Our results predicted an average reduction of 6.1% in the cost per woman tested for HIV, and of 4.2% in the cost per woman on ART or ARV prophylaxis, for every 10% increase in scale.

Another supply-side determinant of efficiency identified in this study was the type of facility. Hospitals were found to be 43% more expensive for HIV testing than clinics, on average. While our data do not allow us to identify the reasons behind this difference, one hypothesis is that relatively sophisticated facilities necessarily provide this simple service at a higher cost than clinics. Another possibility is that the quality of testing services in hospitals is higher with better content, longer duration, and more skilled labor. Our data show that facilities relying more heavily on primary care nurses were 40% less expensive for HIV testing than other using personnel with higher salaries. We did not see a correlation between the quality of services and unitary costs. We found no difference between hospitals and non-hospitals in the cost per woman on ARV prophylaxis or ART.

On the demand side, our results did not suggest a relationship between the complexity of the demand and unitary costs. However, we did observe a strong correlation between demand size and scale. Our results predicted a 6% increase in the number of women on ART for every 10% increase in population size.

In this study, we use instrumental variables as a strategy to estimate the effect of scale on unitary costs of PMTCT services. As mentioned before, previous studies have documented this relationship and found evidence consistent with the existence of economies of scale. However, to our knowledge, this is the first study that uses an econometric strategy to disentangle the endogenous relationship between scale and unitary costs. We instrumented scale with population size at the facility catchment area level. We found that while population and scale are strongly correlated, population and costs are not, which is consistent with the assumptions of our identification strategy. Our results suggest then the presence of economies of scale in the production of PMTCT services in Zimbabwe.

Readers should interpret our results in light of the limitations of the study. First, the data collected on time allocation by staff was self-reported, which has been documented to be biased upwards when compared to more reliable, observation-based methods, such as time-motion studies [13, 14, 22, 41]. Therefore, our approach could lead to an overestimation of unitary costs, however probably not an overestimation of unitary cost variation across facilities, since we used the same method across the whole sample.

Second, our measurement of costs included the major costs categories, namely, staff, ARV drugs, and HIV tests kits. We left out costs associated with recurrent services, such as utilities and fuel, capital costs like buildings and equipment, and costs related to supervision and training. While the relative contribution of these categories to total PMTCT costs has been documented to be less than 10% [13, 14, 33], our estimates provide a lower-bound assessment, nevertheless.

Our indices of process quality focus on completeness or comprehensiveness of services. Although previous studies have found associations between this type of quality proxy and health outcomes and patient's perceptions [36, 37, 38], it captures only one dimension of quality. We did not measure other aspects such as structural quality or health outcomes. Therefore, our results on the effects of process quality on efficiency are not conclusive.

Although the validity of an instrumental variable cannot be fully statistically tested, we tried to verify the two conditions for the instrumental variable model to be valid. We found a strong correlation between population and scale and explored potential channels through which population can affect costs other than scale and did not find any. These results support our choice of instrumental variable.

We found a strong relationship between the scale of services and unitary costs, consistent with the existence of economies of scale and robust after correcting for potential endogeneity bias. However, the policy implication of this finding should not be to provide PMTCT services only in large facilities or in catchment areas with large populations. This interpretation could have undesirable equity consequences on communities in smaller locations. The policy implication should be instead to focus efforts on understanding the differences between inefficient and efficient small facilities—as well as between inefficient and efficient large facilities. In other words, efforts on identifying other determinants of efficiency while accounting for scale effects would be valuable. For example, our results suggest that less sophisticated facilities and staff composition also have efficiency implications. Another policy consequence of the scale effect is for programs to make the appropriate budget planning to adjust for the higher cost per patient expected in smaller facilities. Finally, HIV programs could also consider merging small facilities when doing so could represent gains in efficiency without compromising access to services by the population.

Despite the limitations of the study, our results provide robust cost estimates of the PMTCT program in Zimbabwe using data from a cross-sectional survey of 157 randomly selected health facilities. Furthermore, we found that a large proportion of heterogeneity in unitary costs is explained by scale, which, in turn, is determined by population size in the catchment area. Although our results identify strong associations and control for potential biases, the nature of our data does not allow us to establish robust causal relationships. Program designers nonetheless can draw lessons to improve the efficiency of PMTCT programs without compromising equity.

## Supporting information

S1 File (DOCX)

S2 File (DOCX)

S3 File (DOCX)

S4 File (DOCX)
