# Supplementary material for: Costs and economies of scale in the accelerated program for prevention of mother-to-child transmission of HIV in Zimbabwe
Source: PLoS One. 2020 May 20;15(5):e0231527. doi: 10.1371/journal.pone.0231527 (PMC7239451; doi:10.1371/journal.pone.0231527)
Supplement: S1 File — (DOCX) [file pone.0231527.s001.docx]

# **Supporting Information 1.**

In the Table below, we present a description of the sample of facilities. From the 154 health facilities, the majority were rural health clinics (57 out of 157), and 18 are other type of clinic like mission clinics, industrial clinics, among other types. The first four categories in the table were included in the category “hospital” and the rest in the “non-hospital” category.

**Table A1. Description of the sample: facility types**

| Type of facility | Number of facilities |
| --- | --- |
|  |  |
| District hospital | 9 |
| Mission hospital | 7 |
| Rural health hospital | 7 |
| Private hospital | 5 |
| Local authority | 44 |
| Rural health clinic | 57 |
| Polyclinic | 6 |
| Satellite clinic | 4 |
| Other clinic^[[1]](#footnote-1)^ | 18 |
| Total | 157 |

In Table A2, we present the distribution of staff types. From the total 744 health providers, the majority are primary care nurses and registered general nurses. The rest are sisters in charge and other types of staff as counsellors and health promotion officers.

**Table A2. Description of the sample: staff types**

| Staff type | Frequency | Percent |
| --- | --- | --- |
| Primary care nurse | 325 | 43.68 |
| Registered general nurse | 334 | 44.89 |
| Sister in charge | 31 | 4.17 |
| Other^[[2]](#footnote-2)^ | 54 | 7.26 |
| Total | 744 | 100 |

1. Mission clinics, industrial clinics, private clinics, etc. [↑](#footnote-ref-1)
2. Counsellors, health promotion officers and other support staff [↑](#footnote-ref-2)
