# Supplementary material for: Costs and economies of scale in the accelerated program for prevention of mother-to-child transmission of HIV in Zimbabwe
Source: PLoS One. 2020 May 20;15(5):e0231527. doi: 10.1371/journal.pone.0231527 (PMC7239451; doi:10.1371/journal.pone.0231527)
Supplement: S2 File — (DOCX) [file pone.0231527.s002.docx]

# **Supporting Information 2.**

When are female patients in this facility given advice about their family options or prescribed contraception?

- During antenatal care visits
- After labor and delivery
- During postnatal care visits
- During child immunization visits

In the past month, did this facility offer the following services for pregnant women?

- General health
- HIV testing and counselling
- Antenatal care
- Labor & delivery
- CD4 testing
- Maternal ARV prophylaxis
- Antiretroviral therapy
- Cotrimoxazole for opportunistic infections prophylaxis
- Infant feeding counselling
- Postnatal services
- Family planning

In the past month was ARV or Cotrimoxazole prophylaxis available at this facility for the following patients?

- Maternal ARV prophylaxis
- Infant ARV prophylaxis?
- Cotrimoxazole to HIV+ pregnant women
- Cotrimoxazole to HIV infected children
- Cotrimoxazole to HIV exposed infants
