# Supplementary material for: Costs and economies of scale in the accelerated program for prevention of mother-to-child transmission of HIV in Zimbabwe
Source: PLoS One. 2020 May 20;15(5):e0231527. doi: 10.1371/journal.pone.0231527 (PMC7239451; doi:10.1371/journal.pone.0231527)

# **Supporting Information 3.**

To explore the influence of demand-side characteristics on the variation of unit costs, we analyzed the relationship between demand size and demand complexity and scale. Fig A3 displays scatterplots of the number of women tested per facility and on the number of women initiated on ART/ARV prophylaxis, against three demand-side variables at the catchment area level: HIV prevalence, the percentage of women previously tested as self-reported in the survey, and total population in the catchment area. Across catchment areas, there is large variation in prevalence, which is not strongly correlated with the scale of women tested (correlation coefficient rho= -0.07) nor with the number of women initiated on ART/ARV prophylaxis (correlation coefficient rho= 0.09). The proportion of women already tested for HIV across catchment areas is concentrated between 90% and 100% and is not correlated with number of women tested in 2013 at the facilities (correlation coefficient rho= -0.005), nor with the number of women on treatment and prophylaxis (correlation coefficient rho= 0.04). However, the total population in the catchment area is positively correlated with both the scale of women tested (correlation coefficient rho=0.40*, p<0.01), as well as the number of women initiated on ART/ARV treatment or prophylaxis (correlation coefficient rho=0.64*, p<0.01).

**Figure A3. Correlation between scale and demand-side characteristics per facility-year.**


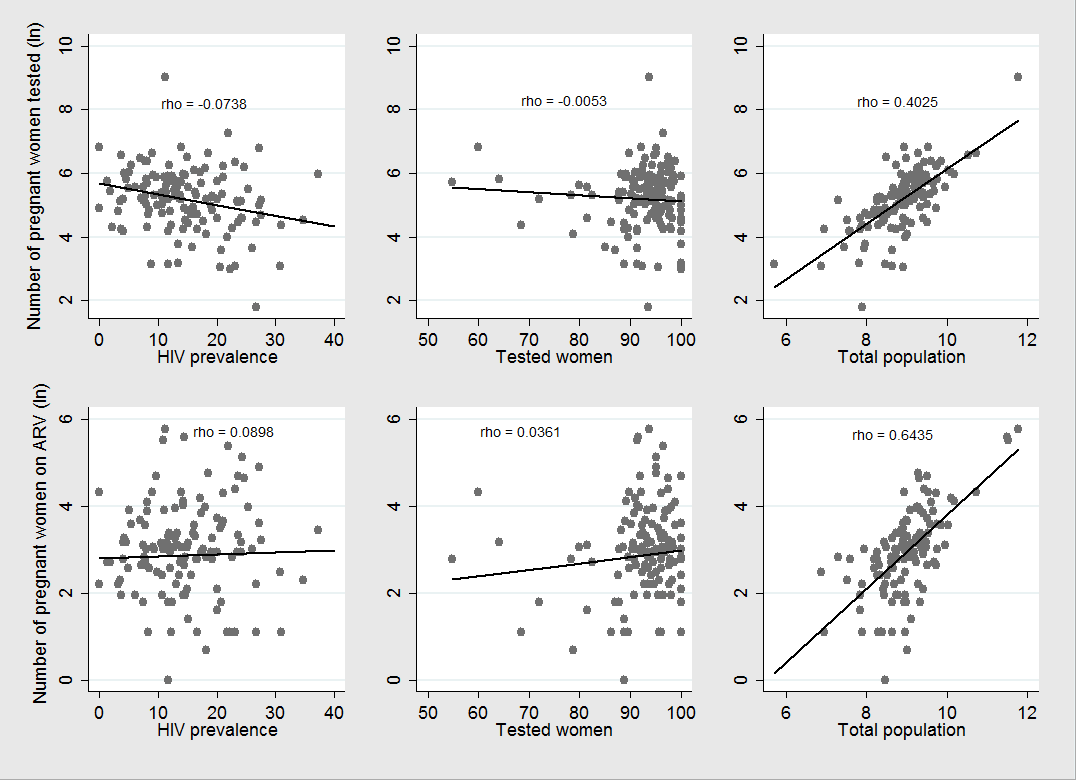

Supplement: S3 File — (DOCX) [file pone.0231527.s003.docx]
