# Supplementary material for: Costs and economies of scale in the accelerated program for prevention of mother-to-child transmission of HIV in Zimbabwe
Source: PLoS One. 2020 May 20;15(5):e0231527. doi: 10.1371/journal.pone.0231527 (PMC7239451; doi:10.1371/journal.pone.0231527)
Supplement: S4 File — (DOCX) [file pone.0231527.s004.docx]

# **Supporting Information 4.**

**Table A4. Correlation matrix of instrumental variable**

|  | Cost of women tested | Cost of women on ART | Population size | Urban=1 | Proportion of higher skilled providers |
| --- | --- | --- | --- | --- | --- |
| Cost of women tested | 1 |  |  |  |  |
| Cost of women on ART | 0.5299* | 1 |  |  |  |
| Population size | -0.1034 | -0.0382 | 1 |  |  |
| Urban=1 | 0.0110 | -0.0069 | 0.7727* | 1 |  |
| Proportion of higher skilled providers | 0.1749 | 0.0479 | 0.3931* | 0.3502* | 1 |

* p<0.01

Note: Higher skilled workers include matron, district nursing officer, sister in charge, mid-wife and senior registered general nurse.
